# Prevalence and Genetic Relationship of Predominant *Escherichia coli* Serotypes Isolated from Poultry, Wild Animals, and Environment in the Mekong Delta, Vietnam

**DOI:** 10.1155/2021/6504648

**Published:** 2021-11-11

**Authors:** Lam Thanh Nguyen, Nguyen Khanh Thuan, Nguyen Thu Tam, Chau Thi Huyen Trang, Nguyen Phuc Khanh, Tran Ngoc Bich, Takahide Taniguchi, Hideki Hayashidani, Ly Thi Lien Khai

**Affiliations:** ^1^Department of Veterinary Medicine, College of Agriculture, Can Tho University, Campus II, 3/2 Street, Ninh Kieu District, Can Tho, Vietnam; ^2^Division of Animal Life Science, Institute of Agriculture, Tokyo University of Agriculture and Technology, 3–5–8 Saiwai-Cho, Fuchu-Shi, Tokyo 183-8509, Japan

## Abstract

Avian pathogenic *Escherichia coli* (APEC) is the main causative agent of avian colibacillosis, which is an important systemic disease of profound economic and clinical consequences for the poultry industry worldwide. In this study, 975 *E. coli* strains were isolated from 2,169 samples collected from cloacal swabs of chickens, in-farm wild animals (ants, geckos, flies, and rats), and environment. The highest proportion of *E. coli* isolation was obtained from chicken cloacal swabs with 71.05% (95% confidence interval (CI) 66.69–75.05%) followed by the proportions of 38.15% (95% CI 35.41–40.97%) and 38.11% (95% CI 34.15–42.24%) from wild animals or environment, respectively. Distribution of O-antigen serotypes of the *E. coli* isolates, including O1, O2, O18, and O78, was determined by PCR. The most predominant serotype was O18 (10.56%) followed by O2 (9.44%), O1 (7.79%), and O78 (6.56%). Of note, serotype O18 was more likely distributed in the examined wild animals, especially in geckos. Polymorphic DNA fingerprints, generated by ERIC-PCR, of representative *E. coli* strains of each serotype revealed genetic heterogeneity of the examined *E. coli,* and O18 was more divergent with 63 clusters formed from 66 isolates. Furthermore, several *E. coli* strains from different sample sources shared high DNA fingerprint relatedness, suggesting that there exists complex transmission of *E. coli* from chickens to wild animals and environment and vice versa in poultry husbandry settings. Although pathotypes of the examined *E. coli* were not determined in this study, our results provided important findings of epidemiological and genetic characteristics of *E. coli* in the Mekong Delta and highlighted the prerequisite of stricter biocontainment to reduce the prevalence and consequences of APEC in poultry production.

## 1. Introduction

*Escherichia coli* (*E. coli*) is a ubiquitous organism in the gastrointestinal microbiota of human and animals. A majority of *E. coli* are nonpathogenic and play an important role in host metabolism, immunology, and nutrition [1]. However, several *E. coli* strains can acquire specific virulence factors and become pathogenic *E. coli* that are capable of causing a wide range of diseases in human and animals [2]. Concerning animal health, avian pathogenic *E. coli* (APEC) is the primary cause of avian colibacillosis; the disease is characterized by multiple systemic syndromes such as colisepticemia, airsacculitis, perihepatitis, pericarditis, swollen-head syndrome, and fatal hemorrhagic septicemia, resulting in high morbidity, mortality, and carcass condemnation [2]. Thus, APEC is responsible for severe economic and clinical consequences to poultry production worldwide [3]. Besides the serious impacts on poultry health, many recent studies have indicated that a subset of APEC might cause zoonotic infections, posing a real threat to public health [4–7].

*E. coli* could be serotyped by differentiating its somatic O (a component of the surface lipopolysaccharide) and H (flagellar) antigens; thus, variability of the O-antigen provides the basis for many serotyping schemes and that becomes the current standard classification of taxonomy and epidemiology of *E. coli* [8, 9]. At present, 181 O-serotypes of *E. coli*, numbered from O1 to O181, have been recognized [8–10], in which several specific O-serotypes of APEC have been frequently confirmed to be closely associated with serious diseases in human and animals. Notably, certain APEC belonging to the serotypes O1, O2, O18, and O78 have been considered the most common pathogenic strains and that is accounted for more than 80% of the APEC isolates [11–13].

Several studies have been carried to investigate the microbiological characterization of APEC, including the distribution of serotypes, attributed virulence, antimicrobial resistance, and phylogeny in several major poultry producing countries such as Spain [14], China [15], and South Korea [16]. However, little knowledge has been elucidated about epidemiological and genetic characteristics of APEC in poultry production in Vietnam where there exists a rapid transition to large and/or industrial-scale poultry enterprises [17]. Therefore, with this background, this study was conducted (i) to determine the prevalence and distribution of common serotypes of APEC in poultry, in-farm wild animals, and environment and (ii) to identify the genetic relationship of *E. coli* strains among these sample sources.

## 2. Materials and Methods

### 2.1. Ethical Approval

All experimental protocols were approved by the Institutional Animal Care and Use Committee of Can Tho University, Vietnam. Capture and dissection of wild animals were ethically performed following the guideline in accordance with the Regulation on Animal Experimentation of Can Tho University.

### 2.2. Sampling Site, Period, and Sample Collection

The sampling procedure was performed in backyards/small poultry farms in Vinh Long Province covering an area of about 1,526 km^2^ with approximately 9.6 million chickens [18], and this province is considered as a central division for poultry production in the Mekong Delta, Vietnam (Supplementary Figure 1). From 2018 to 2020, a total of 2,169 samples including cloacal swabs from healthy chickens, in-farm wild animals including ants, geckos (house gecko, *Hemidactylus frenatus*, flat-tailed house gecko, *Hemidactylus platyurus,* and four-clawed gecko, *Gehyra mutilata*), flies (housefly, *Musca domestica*, and blowfly, Calliphoridae), and rats, and environmental samples were collected in this study. Details of sample types and distribution are described in Supplementary Table 1. All swab samples (cloaca feces; barn floors) were put into a Carry-Blair transport medium (Merck, Germany). Wild animals were trapped and put separately in sterile bags with ventilation holes; feed (250 g) and drinking water (1,000 mL) were collected directly in the flocks. All samples were cooled in an icebox and immediately transported to the laboratory within 24 hours for processing and identification.

In the laboratory, geckos were euthanized by freezing at −20°C in five minutes, while rats were euthanized by using chloroform (Merck, Germany). Both were dissected at room temperature to collect cecal content individually and aseptically. Ants and flies were also inactivated by freezing at −20°C in five minutes, and all bodies were suspended in the enrichment broth of buffered peptone water medium (BPW, Merck, Germany).

### 2.3. *E. coli* Isolation and Identification

Isolation and identification of *E. coli* were performed in accordance with Vietnam National Standard of TCVN 7924-2:2008 [19], equivalent to the standard colony morphology and biochemical identification methods as previously described [20]. Briefly, collected samples were put into the enrichment broth and buffered peptone water medium (BPW, Merck, Germany) and incubated for 24 hours at 37°C. Then, the enriched suspension was inoculated onto MacConkey agar (MC, Merck, Germany) and incubated at 37°C overnight. From each positive sample, ten suspicious *E. coli* colonies were selected for subculture on nutrient agar (NA, Merck, Germany). After incubation for 24 hours at 37°C, those isolates were individually examined for biochemical tests as previously described [21].

### 2.4. DNA Extraction

Total genomic DNA of a single *E. coli* strain was extracted by boiling method. A loopful of a colony on the nutrient agar (NA, Merck, Germany) was dissolved with 1 mL of sterile deionized distilled water and vortexed for 10 s. The suspension was placed on a thermal block at 95°C for 10 min and then was centrifuged at 10,000 rpm for 5 min. The supernatant from this suspension was used as the template for PCR [22].

### 2.5. Serotyping Using Molecular PCR Detection

All the single *E. coli* isolates were serotyped using conventional single PCR with four primer pairs that were previously developed to detect and differentiate each of serotypes O1, O2, O18, and O78 [23]. Each 20 *μ*l PCR reaction mixture comprises 1 *μ*l of genomic DNA template and 18 *μ*l of the 2x colorless Go-Taq master mix that included MgCl_2_, 10x PCR buffer, dNTPs, 10 units of Taq DNA polymerase (Cat #M7132, Promega, Madison, WI, USA), and 0.5 *μ*l of each 10 *μ*M forward and reverse primer. PCR amplification was performed under the following reaction conditions: initial denaturation at 94°C for 5 min, followed by 30 cycles of 95°C for 35 s, 57°C for 30 s, 72°C for 1 min, a final extension at 72°C for 10 min, and then holding at 4°C. The resulting PCR products were visualized on 1.5% tris-acetate-EDTA agarose gels stained with ethidium bromide alongside a 100 bp ladder (Bioline, UK) and photographed under UV transillumination.

### 2.6. ERIC-PCR and Fingerprints Analyses

Representative *E. coli* isolates of a single colony per sample were fingerprinted using the enterobacterial repetitive intergenic consensus- (Eric-) PCR. A set of primers ERIC-1 (5′-ATG TAA GCT CCT GGG GAT TCA C-3′) and ERIC-2 (5′-AAG TAA GTG ACT GGG GTG AGC G-3′) was used to amplify the regions in the bacterial genome positioned between the Eric sequences [24]. The component of the ERIC-PCR reaction mixture was prepared as mentioned above. The ERIC-PCR was performed in a thermocycler under the following condition: initial denaturation at 94°C for 5 min followed by 35 cycles consisting of denaturation at 94°C for 1 min, annealing at 50°C for 1 min, extension at 65°C for 8 min, a final extension step at 65°C for 8 min, and final storage at 4°C. The products of ERIC-PCR were electrophoresed on a 1.5% agarose gel, then stained with ethidium bromide, and visualized by UV transillumination. The image was captured using a gel documentation system for further analysis of DNA fingerprinting.

Polymorphic DNA fingerprint was analyzed using Bionumerics software version 7.0 (Applied Maths, Sint-Martens-Latem, Belgium). Four dendrograms showing clustering of the APEC strains of each serotype were generated based on the averaged similarity of the matrix with the use of the algorithm of the Unweighted Pair Group Method with Arithmetic Mean (UPGMA) analysis and Dice similarity coefficient [24]. A cutoff value of ≥85% similarity coefficient was applied to assign the clusters [25].

### 2.7. Data Execution and Statistical Analysis

Data collection was undertaken using Microsoft Excel. The prevalence proportion (expressed as a percentage) of *E. coli* isolation was determined, and its confidence intervals (CI) were calculated as 95% binomial proportions representing Wilson score intervals. Statistical analyses, graphing, and visualization were performed through computing platform R version 4.0.1 [26], contributed packages ggplot2 [27], binom [28], and epiR [29].

## 3. Results

### 3.1. Prevalence of *E. coli*

**Figure 1:**
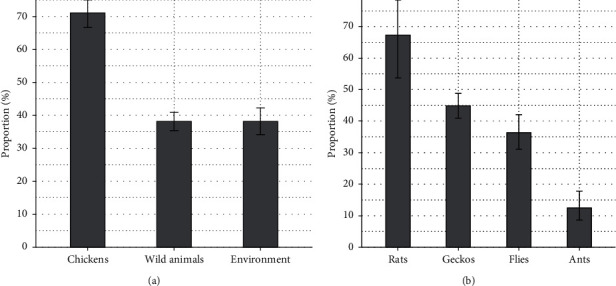
Bar graphs showing the proportion of *E. coli* isolated from cloacal swabs of chickens, wild animals, and environment (a) and proportion of *E. coli* isolated from ants, geckos, flies, and rats (b). The error bar indicates 95% confidence interval.

A total of 975 *E. coli* strains were isolated from 2,169 samples collected from cloacal swabs of chickens, in-farm wild animals (ants, geckos, flies, and rats), and environment. The proportions and the CIs of *E. coli* isolates per sample are presented in Figure 1. *E. coli* was isolated from 319 of 449 (71.05% (95% CI 66.69–75.05%)) cloacal swab samples of chickens, which was significantly higher than the number of *E. coli* either isolated from wild animals 446 of 1,169 (38.15% (95% CI 35.41–40.97%)) and environment 210 of 551 (38.11% (95% CI 34.15–42.24%)), respectively (Figure 1(a)). Within the studied wild animals, the number of *E. coli* isolated from rats was 35 of 52 (67.30% (95% CI 53.76–78.48%)) that was the highest proportion in comparison with geckos 278 of 620 (44.84% (95% CI 40.97–48.77%)), flies 108 of 297 (36.36% (95% CI 31.10–41.98%)), and ants 25 of 200 (12.50% (95% CI 8.61–17.80%)), respectively (Figure 1(b)).

### 3.2. Distribution of Serotypes O1, O2, O18, and O78 in *E. coli* Isolates

**Figure 2:**
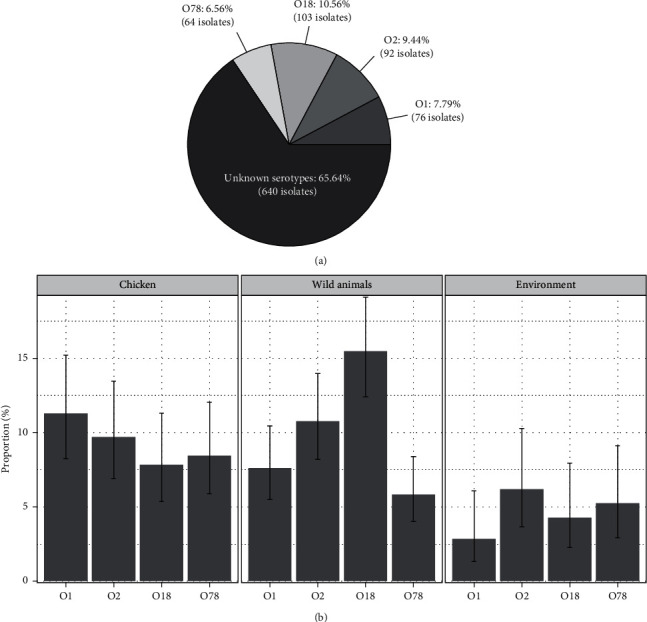
A pie chart showing the distribution of serotypes O1, O2, O18, and O78 per all *E. coli* isolates (a) and bar graphs showing proportions of serotypes O1, O2, O18, and O78 per chicken, wild animals, and environment (b). The error bar indicates 95% confidence interval.

All 975 *E. coli* strains were serotyped with O1, O2, O18, and O78 using conventional PCR (Supplementary Figure 2). The distribution of the examined O-serotypes is shown in Figure 2. Proportions of *E. coli* belonging to the serotypes O1, O2, O18, and O78 were 7.79%, 9.44%, 10.56%, and 6.56%, respectively. A large remaining proportion (65.64%) of *E. coli* was unknown serotypes (Figure 2(a)). In chickens, the distribution of O1, O2, O18, and O78 *E. coli* was almost equivalent with the proportions of 11.29% (95% CI 8.26–15.22%), 9.72% (95% CI 6.93–13.46%), 7.84% (95% CI 5.36–11.31%), and 8.46% (95% CI 5.88–12.03%), respectively. Similarly, the presence of these four serotypes was comparable in the environment samples with proportions ranging from 2.86% (O1) to 6.19% (O2) (Figure 2(b)). Remarkably, O18 *E. coli* was accounted for a relatively large proportion of 15.47% (95% CI 12.41–19.12%) of *E. coli* isolated from wild animals, and the majority of the O18 *E. coli* were isolated from in-farm geckos (46.39%), 45 O18 *E. coli* isolates from geckos out of total 97 O18 *E. coli* isolates in all collected samples (data not shown).

### 3.3. Genomic DNA Fingerprinting and Genetic Relationship of O1, O2, O18, and O78 *E. coli*

**Figure 3:**
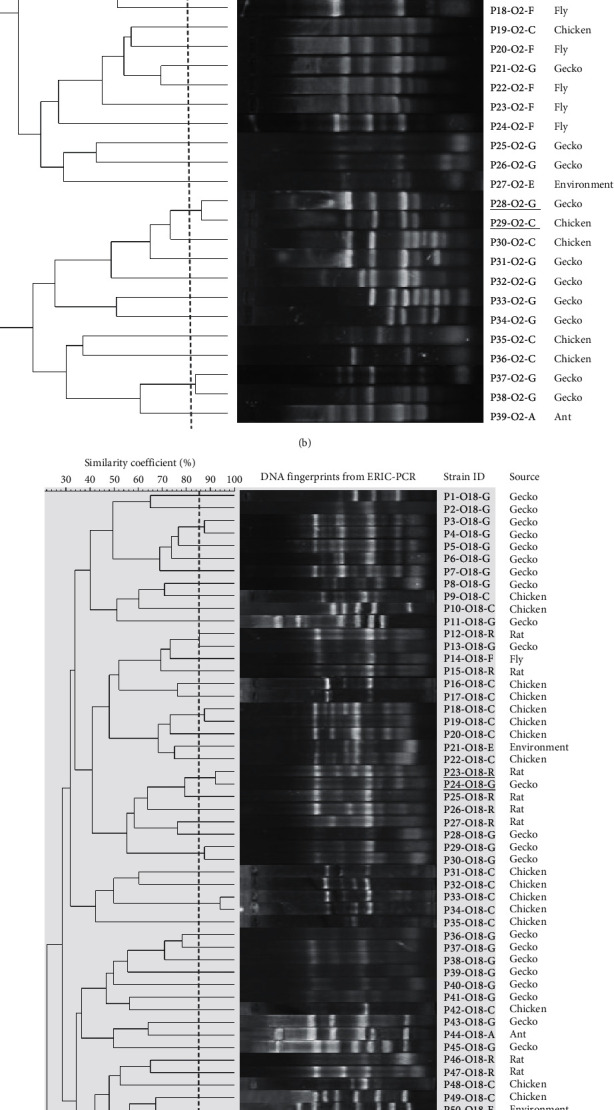
Dendrograms and genomic DNA fingerprints of *E. coli* isolates that belong to the O1 (a), O2 (b), O18 (c), and O78 (d). The results were produced from ERIC-PCR and Bionumerics software version 7.0. The dashed vertical line indicates the cutoff value ≥85% genetic similarity coefficient. *E. coli* strains that were isolated from different sample sources and shared ≥85% genetic similarity coefficient were underlined.

Representative *E. coli* isolates of serotypes O1 (42 isolates), O2 (39 isolates), O18 (66 isolates), and O78 (39 isolates) were selected based on sample types and space-time sources for ERIC-PCR to identify genetic divergence and relationship among the *E. coli* isolates. Four dendrograms generated from genomic fingerprinting of *E. coli* isolates belonging to the examined serotypes were, respectively, described in Figures 3(a)–3(d). These results showed that DNA fingerprinting patterns of *E. coli* were heterogeneous, which was characterized by allocation of multiple genetic subclusters per serotype: O1 (38 clusters out of 42 isolates), O2 (35 clusters out of 39 isolates), O18 (64 clusters out of 66), and O78 (35 clusters out of 39 isolates) and all these subclusters were grouped into two (O78) to three (O1, O2, and O18) major clusters. Importantly, within the same serotype, several *E. coli* strains isolated from different sample sources shared high genetic similarity (≥85%) and grouped together into distinct subclusters, for example, O1 serotypes (P4–O1–C/P5–O1-G, P18–O1–C/P19–O1-E, and P37–O1–C/P38–O1-E); O2 serotype (P9–O2–C/P10–O2-E, P12–O2–C/P13–O2-E, and P28–O2-G/P29–O2–C), O18 serotype (P23–O18-R/P24–O18-G), and O78 serotype (P17–O78-E/P18–O78–C and P35–O78–C/P36–O78-E).

## 4. Discussion

Avian pathogenic *E. coli* is the major causative agent of avian colibacillosis that is considered one of the most important bacterial diseases in the global poultry industry due to its substantial economic impacts [2, 30]. In addition to causing serious consequences on poultry health and production, several APEC strains have been determined as potential zoonotic pathogens [4–7]. Therefore, comprehensive knowledge of biological and epidemiological characteristics of APEC is critical to reducing the incidence of avian colibacillosis in poultry production and threats to human health. In this study, we first carried out isolation and identification of *E. coli* collected from chickens, wild animals, and environment. Then, the obtained *E. coli* isolates were serotyped using PCR targeting O-antigen genes of the notable O1, O2, O18, and O78 serotypes, and finally, the genetic relationship of these *E. coli* was examined using DNA fingerprints generated from ERIC-PCR.

A total of 975 *E. coli* strains were isolated from 2,169 samples (44.95% isolation rate) from chickens, wild animals, and environment. The highest isolation rate was from chicken samples (71.05% (95% CI 66.69–75.05%)) as compared with the wild animals and environment samples (Figure 1). Among wild animals, relatively high isolation rates were also obtained from rats (67.31% (95% CI 53.76–78.48%)) and geckos (44.84% (95% CI 40.97–48.77%)). This result is consistent with the common biological plausibility that *E. coli* coexist in the gastrointestinal tract as harmless commensal symbionts in warm-blooded animals [1]. Nevertheless, the high prevalence of *E. coli* recovered from chickens, one of the major reservoirs of APEC, and in-farm wild animals might be an issue for poultry husbandry since a subset of otherwise commensal *E. coli* strains derived from the animals could be pathogenic *E. coli* and potentially implicate localized or systemic infections when birds suffer from immune suppression, stress, or injury [2].

A few studies have been done to elucidate microbiological characteristics of *E. coli* existing in poultry production in Vietnam. Most studies focused on investigating virulence attributes and/or antibiotics resistance of *E. coli* strains obtained from either poultry host species and environment [31, 32]; however, information about the distribution of serotypes of such *E. coli* remains elusive. In this study, all 975 *E. coli* strains were subjected to serotyping using conventional PCR. As literature review, APEC serotypes O1, O2, O18, and O78 were targets for the present serotyping since it has been widely reported that *E. coli* strains in these serotypes are the most common APEC pathotypes causing colibacillosis in animals and human [7, 33]. A total of 335 *E. coli* out of 975 (34.35%) *E. coli* strains were defined as belonging to the serotypes O1, O2, O18, and O78 (Figure 2(a)). These serotypes were distributed at almost equivalent proportions among chickens, wild animals, and environment (Figure 2(b)). This is in line with previous studies showing that the prevalence of *E. coli* ranges from 9.52% to 36.73% in all age groups of chickens [34]. Remarkably, serotype O18 was more likely predominant with the highest proportion at 10.56% followed by O2 (9.44%), O1 (7.79%), and O78 (5.56%). The higher proportion of the O18 *E. coli* gives greater consideration since certain O18 APEC strains have been reported to have zoonotic importance of causing urinary tract infections and meningitis in humans [35]. On the other hand, 45 isolates, out of the total 97 (46.39%) O18 *E. coli* isolates recovered from all samples, were derived from geckos. Several previous studies also raise concerns about geckos' role in disease transmission where the species can be either natural reservoirs and transmitters for pathogenic bacteria such as APEC and *Salmonella* [16, 36–38]. It is also noteworthy to mention that more than half (65.64%) of the *E. coli* isolates were not assigned their serotypes. There might be important *E. coli* strains linked to other APEC pathotypes and should be included for further serotyping targets in order to provide explicit information about the population characteristics of APEC.

ERIC-PCR was employed to determine molecular fingerprints of examined *E. coli* since this technique is reproducible and cost-effective and has been widely used to evaluate the genetic relatedness of bacteria from different origins [39]. Dendrograms generated from ERIC-PCR fingerprints of 42 O1 isolates, 39 O2 isolates, 66 O18 isolates, and 39 O78 isolates showed that *E. coli* present in the settings of poultry husbandry were highly divergent. Multiple subclusters were assigned within each serotype population (O1 : 38 clusters, O2 : 35 clusters, O18 : 64 clusters, and O78 : 35 clusters). The number of subclusters per serotype in this study is obviously higher than that observed in previous investigations using the same method [40–42]. More heterogeneity of clustering patterns in our study might result from a larger number of isolates or variety of sample sources included in our analysis or might be due to the more rapid evolution of *E. coli* in the Mekong Delta [31, 32]. As a result, this study reaffirms that *E. coli* existing in the poultry husbandry in Vietnam has undergone large and rapid genetic diversification.

This study places greater emphasis on inferring genetic relationship and cross-contamination potentials of *E. coli* between different host species and environment on the basis of DNA fingerprints generated from ERIC-PCR. It is evident from the four O1, O2, O18, and O78 fingerprint dendrograms that several *E. coli* strains collected from chickens, wild animals, and environment were grouped in the same subclusters with high genetic similarities (Figures 3(a)–3(d)). This result suggests that there might be cross transmission of *E. coli* between different sources in the poultry husbandry setting. Previously, several studies also demonstrated that fecal contamination from poultry is the primary dissemination and transmission source of *E. coli* to environment and surrounding animals [43, 44]. In fact, from our empirical observation, almost all small/backyard farms in the Mekong Delta have relatively poor hygiene practices, and such wild animals as geckos, rats, flies, and ants commonly exist in poultry farms. Thus, cross transmission is almost certain. This study suggests that monitoring *E. coli* as well as APEC contamination in environment and in-farms wild animals could be considered as an indicator of interspecies barrier transposition in poultry production. In addition, the observed high prevalence of *E. coli* belonging to the O1, O2, O18, and O78 serotypes from wild animals in poultry farms is also concerning since these species also play important roles in food-borne disease transmission but they are usually neglected in routine surveillance programs [36, 38].

This study successfully examined in part the population structure of *E. coli* with the four common APEC serotypes and provided genetic evidence for cross transmission of these *E. coli* serotypes between different sources in the poultry husbandry. However, there are some inevitable limitations in the present study; for example, virulence, pathotypes, and antibiotics resistance characteristics of the obtained *E. coli* were not examined or a large proportion of *E. coli* was not serotyped. Nevertheless, this study added significant information on biological characteristics and epidemiological dynamics of *E. coli* which is necessary for better control of avian colibacillosis and prevention of the threat of zoonotic diseases caused by APEC.

## 5. Conclusion

*E. coli* was predominantly isolated from small/backyard poultry farms. Serotypes O1, O2, O18, and O78 were accounted for a high proportion of the *E. coli* population and O18 was the predominant serotype among the examined *E. coli*. Chickens, in-farms geckos, and rats were identified as the major sources of dissemination and transmission of *E. coli*. Polymorphic DNA fingerprint analyses revealed that APEC from different sources of poultry husbandry exhibit large genetic heterogeneity and closed genetic relationship. Therefore, this study provides important information about epidemiological and genetic characteristics of *E. coli* and emphasizes the necessity of stricter biocontainment in poultry production for prevention and control of avian colibacillosis caused by APEC.

## Data Availability

All data generated or analyzed during this study are included in this published article and its supplementary information files.

## Supplementary Materials

Supplementary Materials
*Supplementary Table 1*. Summary of sample types and their distribution. *Supplementary Figure 1*. Geographical map showing the location of sampling site: Vinh Long Province (black) is situated in the center of the Mekong Delta (grey) on the Vietnam map. *Supplementary Figure 2*. The PCR products amplified using the allele-specific genes of O1 (3-A), O2 (3-B), O18 (3-C), and O78 (3-D) serotypes. Lane M: 100 bp DNA ladder; lane P: positive control; lane N: negative control (distilled water as template); lane 1 to lane 6: DNA samples of *E. coli* tested. Arrows indicate sizes of amplified DNA fragments as follows: 263 bp (O1), 355 bp (O2), 459 bp (O18), and 623 bp (O78). .
