# Supplementary material for: Prevalence and Genetic Relationship of Predominant Escherichia coli Serotypes Isolated from Poultry, Wild Animals, and Environment in the Mekong Delta, Vietnam
Source: Vet Med Int. 2021 Nov 11;2021:6504648. doi: 10.1155/2021/6504648 (PMC8601835; doi:10.1155/2021/6504648)
Supplement: Supplementary Materials — Supplementary Table 1. Summary of sample types and their distribution. Supplementary Figure 1. Geographical map showing the location of sampling site: Vinh Long Province (black) is situated in the center of the Mekong Delta (grey) on the Vietnam map. Supplementary Figure 2. The PCR products amplified using the allele-specific genes of O1 (3-A), O2 (3-B), O18 (3-C), and O78 (3-D) serotypes. Lane M: 100 bp DNA ladder; lane P: positive control; lane N: negative control (distilled water as template); lane 1 to lane 6: DNA samples of E. coli tested. Arrows indicate sizes of amplified DNA fragments as follows: 263 bp (O1), 355 bp (O2), 459 bp (O18), and 623 bp (O78). . [file 6504648.f1.pdf]

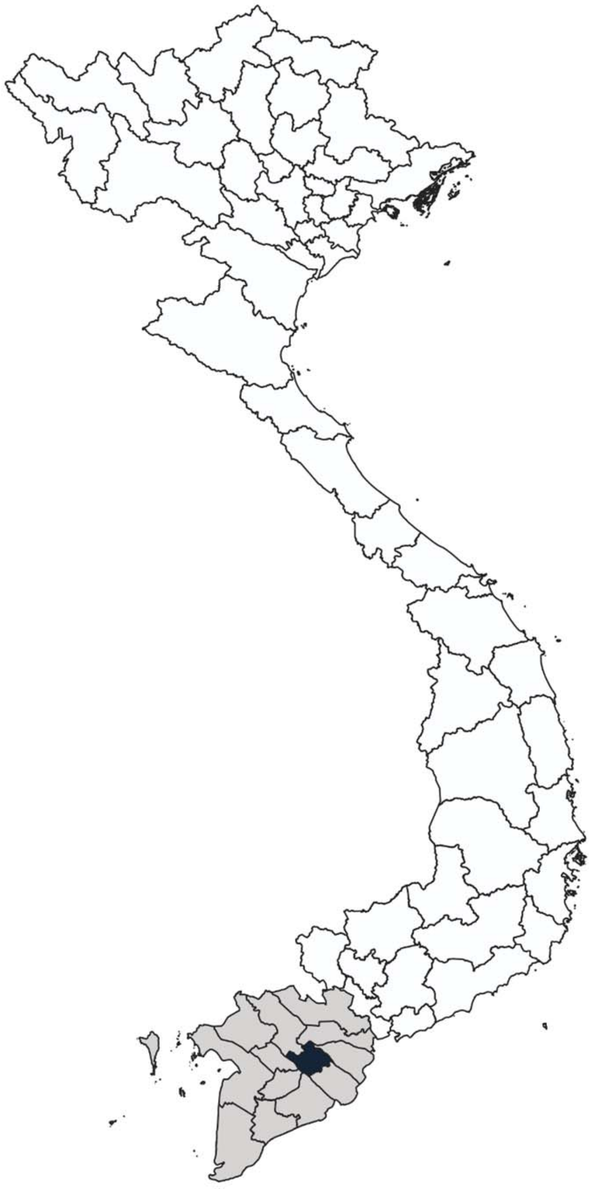

Supplementary Figure 1. Geographical map showing the location of sampling site – Vinh Long province (black) is situated in the centre of Mekong delta (grey) on the Vietnam map.

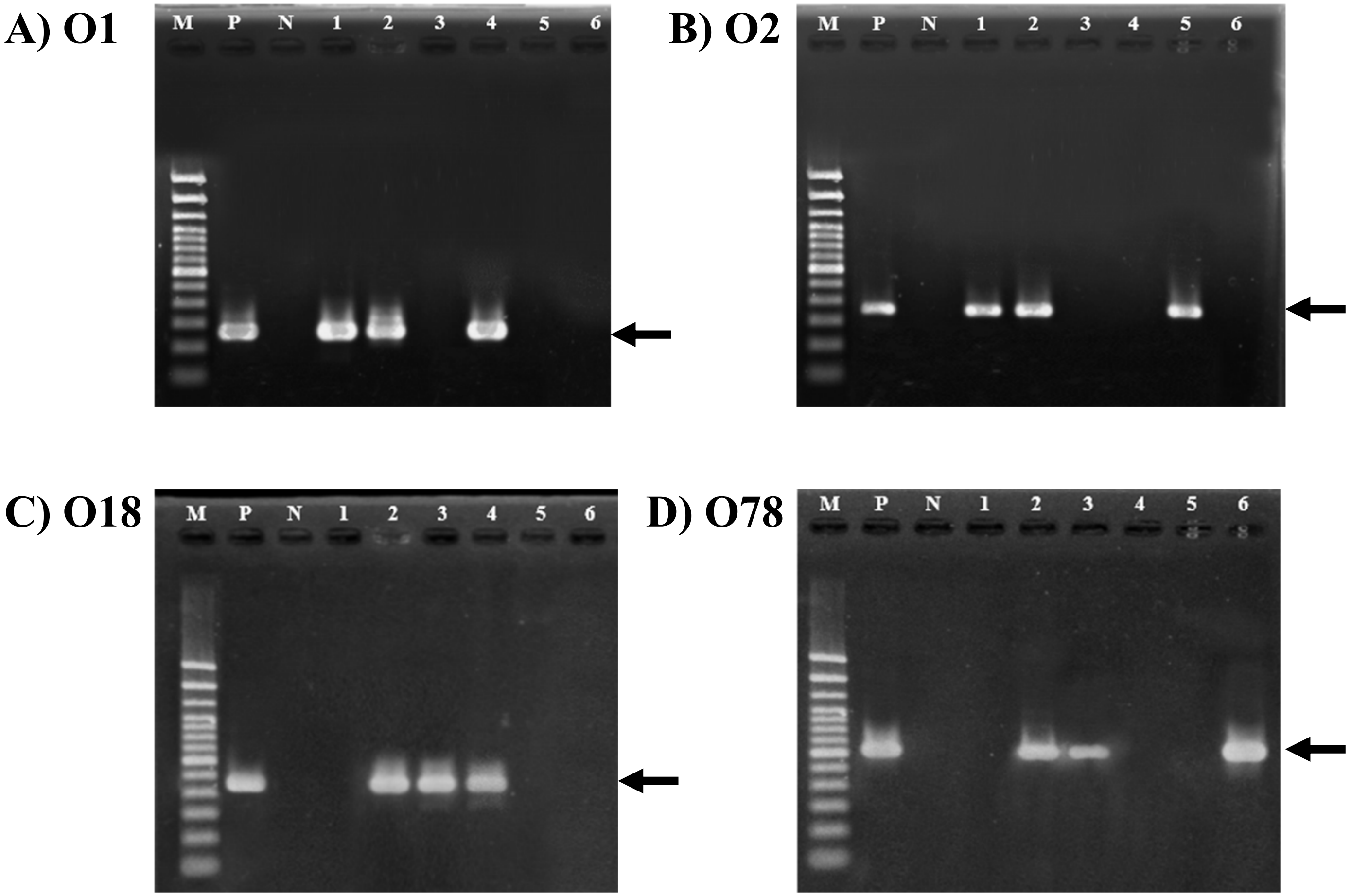

Supplementary Figure 2. The PCR products amplified using the allele-specific genes of O1 (**3-A**), O2 (**3-B**), O18 (**3-C**) and O78 (**3-D**) serotypes. Lane M: 100-bp DNA ladder; Lane P: Positive control; Lane N: Negative control (distilled water as template); Lane 1 to Lane 6: DNA samples of *E. coli* tested. Arrows indicate sizes amplified DNA fragments as followed 263 bp (O1), 355 bp (O2), 459 bp (O18) and 623 bp (O78).

Supplementary Table 1. Summary of sample types and its distribution

Districts	Sample types	Type samples	No. of samples
A	Chicken	Cloacal swab	240
	Wild animals:		
	Ants	Body	120
	Geckos	Cecal contents	250
	Flies	Body	137
	Rats	Cecal contents	0
	Environment	Barn floor, drinking water and feed	300
B	Chicken	Cloacal swab	209
	Wild animals:		
	Ants	Body	80
	Geckos	Cecal contents	370
	Flies	Body	160
	Rats	Cecal contents	52
	Environment	Barn floor, drinking water and feed	251
	**Total**		**2,169**
